# A novel biomarker of laminin turnover is associated with disease progression and mortality in chronic kidney disease

**DOI:** 10.1371/journal.pone.0204239

**Published:** 2018-10-01

**Authors:** Signe Holm Nielsen, Daniel Guldager Kring Rasmussen, Susanne Brix, Anthony Fenton, Mark Jesky, Charles J. Ferro, Morten Karsdal, Federica Genovese, Paul Cockwell

**Affiliations:** 1 Nordic Bioscience, Biomarkers and Research, Herlev, Denmark; 2 Department of Biotechnology and Biomedicine, Technical University of Denmark, Kgs. Lyngby, Denmark; 3 University of Southern Denmark, Institute of Molecular Medicine, Cardiovascular and Renal Research, Institute of Clinical Research, Odense, Denmark; 4 Department of Renal Medicine, Queen Elizabeth Hospital, Birmingham, United Kingdom; 5 College of Medical and Dental Sciences, University of Birmingham, Birmingham, United Kingdom; Hospital Universitario de la Princesa, SPAIN

## Abstract

**Background:**

Patients with chronic kidney disease (CKD) have increased risk of development of end-stage renal disease (ESRD) and early mortality. Fibrosis is the central pathogenic process in CKD and is caused by dysregulated extracellular matrix (ECM) remodeling. The laminin γ1 chain (LAMC1) is a core structural protein present in the basement membrane of several organs, including the kidneys. We hypothesized that dysregulation of LAMC1 remodeling could be associated with a higher risk of adverse clinical outcomes in patients with CKD.

**Methods:**

A novel immunoassay targeting LG1M, a specific MMP-9-generated neo-epitope fragment of LAMC1, was developed and used to measure the levels of the fragment in urine and serum from 492 patients from the Renal Impairment in Secondary Care (RIISC) study, a prospective cohort of patients with high-risk CKD. Patients were monitored for a median follow-up time of 3.5 years. Associations between serum and urine LG1M levels and progression of CKD at 12 months were assessed by a multivariable logistic regression model. The association with ESRD or mortality was assessed by Kaplan-Meier survival curves and Cox proportional hazards regression.

**Results:**

Forty-six (11%) of the 416 patients who reached 12-month follow-up had progression of CKD; during the study follow-up, 125 patients (25.4%) developed ESRD and 71 patients (14.4%) died. Serum and urine levels of LG1M correlated with baseline eGFR (r = -0.43, p<0.0001 and r = -0.17, p = 0.0002, respectively). Serum levels of LG1M were higher in patients with one-year progression of CKD compared to those who did not progress (p<0.01). Baseline serum levels of LG1M were associated with development of ESRD (HR 3.2, 95% CI 1.99–5.2 for patients in the highest LG1M tertile compared to patient in the lowest tertile). Baseline urinary levels of LG1M (uLG1M) were significantly associated with mortality (HR 5.0, 95% CI 2.8–8.9, p<0.0001 for patients in the highest LG1M tertile compared to patients in the lowest tertile). Urine LG1M was retained in the model for prediction of mortality (HR per standard deviation of uLG1M: 1.01, 95% CI 1.00–1.02, p = 0.001).

**Conclusions:**

LG1M, a marker of basement membrane remodeling, is increased in serum and urine of patients with CKD and levels are associated with one-year disease progression, development of ESRD, and mortality.

## Introduction

Patients with chronic kidney disease (CKD) have an increased risk of developing end-stage renal disease (ESRD)[1] and an increased risk of mortality[2] compared to individuals with normal kidney function. Renal fibrosis is a hallmark of CKD and it is caused by an imbalance between extracellular matrix (ECM) formation and degradation[3]. Both glomerular and tubulo-interstitial ECM are remodeled during the development of renal fibrosis; central to this process is the glomerular and tubular basement membrane, an intricate protein meshwork which separates epithelium, glomerular mesangium, and endothelium from the connective tissue[4,5].

Laminins are intertwined, heterotrimeric glycoproteins containing an α, β, and γ chain[6]; and are a major component of the basement membrane[7]. The known binding partners for laminin include integrins[8], nidogen[9,10] and dystroglycan[11]. Laminin is a substrate of matrix metalloproteinases (MMP)-9, an enzyme that is involved in ECM remodeling and highly upregulated in renal fibrosis.[12–14].

The laminin gamma-1 chain (LAMC1) is the most widely expressed laminin chain and is found in laminin-1-4 and 6–11, which are present in every basement membrane except in the central nervous system and in the retina[15,16]. Fragments of LAMC1 in the serum and urine could reflect the accelerated remodeling of both glomerular and tubular basement membrane, and other organs, and function as surrogate markers of ongoing fibrosis.

We developed a specific immunoassay for the detection of the neo-epitope peptide LG1M, a fragment of LAMC1 generated by MMP-9. We then measured the concentration of LG1M in serum (sLG1M) and urine (uLG1M) from patients with CKD at high risk of progression, and assessed its association with adverse outcomes including CKD progression, development of ESRD and mortality.

## Subjects and methods

### Reagents

All reagents used in the experiments were high standard chemicals from Merck (Whitehouse Station, NJ, USA) and Sigma Aldrich (St. Louis, MO, USA). The synthetic peptides used for immunization and assay development were 1) Immunogenic peptide: KLH-GGC-LNRKYEQAKN, 2) Screening peptide: Biotin-LNRKYEQAKN, 3) Standard peptide: LNRKYEQAKN, and 4) Elongated peptide: LNRKYEQAKNI. All synthetic peptides were purchased from the Chinese Peptide Company, Beijing, China.

### Assay development

Generation of the monoclonal antibody, clone characterization, protocol, technical evaluation and biological validation are detailed in supplementary materials and methods.

### Study population

A total of 492 participants from the Renal Impairment in Secondary Care (RIISC) cohort (NCT01722383) were included in this study. The RIISC study is a prospective observational cohort study designed to identify determinants of adverse outcomes in CKD. Detailed methodology has been previously described[17].

Briefly, patients from nephrology clinics in Birmingham (UK) with non-dialysis high risk CKD were invited to participate in the study. High risk CKD was as defined by the UK National Institute for Health and Care Excellence 2008 CKD guideline[18], and comprised one or more of: estimated glomerular filtration rate (eGFR) < 30 mL/min/1.73m^2^, or eGFR 30–59 mL/min/1.73m^2^ with a decline of ≥5 mL/min/1.73m^2^/year or ≥10 mL/min/1.73m^2^/5 years, or a urinary albumin:creatinine ratio (ACR) ≥70 mg/mmol on three occasions. All patients who received immunosuppression for immune-mediated renal disease and patients who had started renal replacement therapy (RRT) were excluded. All participants had demographic, clinical and laboratory data collected at recruitment and during follow up. Patients consented to follow-up for 10 years, or until death or initiation of renal replacement therapy (RRT). Ethical approval was granted by South Birmingham Local Research Ethics Committee (reference: 10/H1207/6). All patients provided written consent and the study was conducted in accordance with the Declaration of Helsinki. Data and samples from the six-month study visit were used for this analysis. The first 492 patients recruited into the study who had both serum and urine available from their six-month visit were included; these visits occurred between April 2011 and September 2014. The last follow-up data collection occurred on 31^st^ of January 2017 and patients who had not reached a study end point were censored on this date.

The primary outcomes of interest were 1) One-year progression, defined as a decline in eGFR of ≥ 30% or initiation of RRT within 12 months; 2) development of ESRD, defined as the initiation of RRT; 3) death.

Initiation of RRT was captured through the use of a local electronic database, and death was captured through electronic patient records linked to the Office of National Statistics.

### Laboratory analyses

Serum and urine were processed immediately after collection according to pre-defined standard operating procedures and stored at -80°C until analysis. Biochemistry results from the local clinical laboratory were obtained from tests performed in accordance with the current standard of care.

Serum creatinine measurements were performed on a Roche Modular Analyser using a blank rated compensated Jaffe reaction, while urinary ACR was measured using the ADVIA 1800 Chemistry System (Bayer HealthCare). eGFR was estimated using the creatinine-based CKD-EPI equation[19]. C-reactive protein (CRP) was measured using the Full Range C-Reactive Protein Kit on a SPA automated PLUS turbidimeter (The Binding Site Group Ltd, UK). The normal range for CRP is between 0·1 and 9 mg/L, with 90 percent below 3 mg/L.[20] Serum kappa (κ) and lambda (λ) free light chain (FLC) concentrations were measured by nephelometry on a Dade-Behring BNII Analyser (Siemens AG, Erlangen, Germany) using particle enhanced high-specificity homogenous immunoassays (Freelite: The Binding Site Group Ltd, Birmingham, UK). The normal reference ranges for serum FLC concentrations have previously been described as κ: 3.3–19.4 mg/L and λ: 5.7–26.3 mg/L, with the same assay sensitivity being demonstrated as <1 mg/L. κ FLC and λ FLC concentrations were combined to calculate the serum FLC (cFLC) concentrations. All uLG1M measurements are normalized to urine creatinine levels measured by QuantiChrom Creatinine Assay Kit (Bioassay System).

### Statistical analyses

Statistical analysis was carried out using MedCalc (Ostend, Belgium) and GraphPad Prism version 7 (GraphPad Software, Inc., CA, USA). Baseline characteristics stratified per tertiles of sLG1M and uLG1M were presented as number (frequency) and percentage for categorical variables and as median (interquartile range) for continuous variables. Differences between tertiles were assessed using Pearson’s chi-square for categorical variables, and ANOVA (parametric) or Kruskal-Wallis test (non-parametric) for continuous variables.

A linear regression analysis with 95% confidence interval was performed for sLG1M and eGFR; and uLG1M and eGFR.

The association between sLG1M and uLG1M and one-year progression was assessed by logistic regression and presented as odds ratio (OR) with 95% confidence intervals (CI). The association between sLG1M and uLG1M and ESRD and death was assessed by Kaplan-Maier survival curves for tertiles of sLG1M and uLG1M. Cox proportional hazard regression (hazard ratios (HR) with 95% CI) was used to generate time-to-event data for development of ESRD and death, and analyze the association between baseline characteristics and risk of ESRD and death. Clinical relevant confounding factors (eGFR and ACR) together with other variables with a p<0.1 on univariable analysis were included as candidate variables in the multivariable analysis.

For all statistical analysis performed, a p-value below 0.05 was considered significant. Asterisks indicate the following: *: p<0.05; **: p<0.01; ***: p<0.001.

## Results

### Technical validation

The LG1M assay was highly specific for the target sequences and was approved as a technically robust assay. A more detailed description of the results of the technical validation of the assay can be found in the supplementary results.

### Baseline characteristics of study subjects

Of the 492 RIISC participants included in this study, one had an insufficient amount of urine available for analysis, and therefore uLG1M was measured in 491 participants.

The cohort was 61.3% male, median age was 62.5 years (IQR 50–76), and ethnicity was 72.4% white, 17.7% south Asian, 8.7% black and 1% other. The primary renal diagnosis was ischaemic/hypertensive nephropathy in 25.4%, diabetic kidney disease in 9.8%, glomerulonephritis in 16.9%, polycystic kidney disease in 5.9%, and other or uncertain etiology in 42%. Median eGFR was 26.5 mL/min/1.73 m^2^ (IQR 19.4–34.6), median ACR was 31.9 mg/mmol (IQR 6.1–126.2), median serum LG1M was 21.5 ng/mL (IQR 11.3–36.1) and median urinary LG1M/creatinine was 34.6 mg/mL (24.0–47.2). Baseline demographic, social, clinical, and laboratory data for the study participants stratified by sLG1M and uLG1M tertiles are presented in Tables 1 and 2, respectively.

**Table 1:** Baseline demographic, social, and clinical characteristics of the cohort, divided into sLG1M tertiles. Data are presented as number (%) for categorical variables, while continuous variables are presented as median (IQR). DM = diabetes mellitus; COPD = chronic obstructive pulmonary disease; CVD = cerebrovascular disease; IHD = ischaemic heart disease; PVD = peripheral vascular disease; CCI = Charlson’s comorbidity index; IMD = index of multiple deprivation; BMI = body mass index; PWV = pulse wave velocity; PP = pulse pressure; CKD-EPI = Chronic Kidney Disease Epidemiology Collaboration; eGFR = estimated glomerular filtration rate; ACR = albumin creatinine ratio; CRP = C-reactive protein; cFLC = combined free light chains.

	sLG1M tertiles			p-value
	1 [8.5–11.4]	2 [17.4–25.1]	3 [36.1–62.1]	
**n**	164	164	164	
**Age (years)**	57 (44–74)	64 (54–76)	70 (58–78)	**<0.001**
**Sex (male)**	101 (62%)	94 (57%)	106 (65%)	0.36
**EthnicityWhite** **South Asian** **Black** **Other**	118 (72%) 26 (16%) 18 (11%) 2 (1%)	120 (73%) 29 (18%) 14 (8%) 1 (1%)	118 (72%) 32 (20%) 11 (7%) 2 (1%)	0.99 0.73 0.42 0.82
**Primary renal diagnosis** **Ischaemia/Hypertensive nephropathy** **Diabetes** **Glomerulonephritis** **Polycystic kidney disease** **Other/uncertain**	31 (22%) 10 (7%) 39 (27%) 15 (10%) 48 (34%)	45 (30%) 14 (9%) 21 (14%) 10 (7%) 59 (40%)	3149 (31%) 24 (15%) 23 (15%) 4 (3%) 57 (36%)	0.12 **0.04** **0.03** **0.04** 0.54
**Co-morbidities** **Malignancy** **DM** **COPD** **CVD** **IHD** **PVD**	24 (18%) 41 (32%) 15 (12%) 16 (12%) 26 (20%) 8 (6%)	24 (13%) 61 (32%) 17 (9%) 20 (11%) 44 (24%) 21 (11%)	22 (11%) 77(38%) 27 (14%) 16 (8%) 39 (19%) 20 (10%)	0.94 0.014 0.12 0.74 0.09 **0.04**
**Smoking status (current smoker)**	27 (16%)	21 (13%)	17 (10%)	0.27
**IMD Score**	29 (15–44)	27 (15–44)	32 (20–47)	0.06
**BMI (kg/m**^**2**^**)**	28 (25–31)	29 (25–33)	30 (26–35)	**0.01**
**Systolic blood pressure (mmHg)**	119 (110–131)	124 (115–139)	128 (115–145)	**<0.001**
**Diastolic blood pressure (mmHg)**	76 (68–83)	75 (67–83)	73 (66–80)	0.07
**PWV (mmHg)**	9.4 (8.0–10.7)	9.6 (7.9–11.1)	10.0 (8.4–11.8)	**0.008**
**PP (mmHg)**	41 (35–55)	48 (37–62)	55 (43–69)	**<0.001**
**Cystatin C (mg/L)**	1.9 (1.5–2.6)	2.5 (2.0–3.0)	2.8 (2.2–3.4)	**<0.001**
**CKD-EPI eGFR (mL/min/1.73m**^**2**^**)**	32 (24–43)	27 (20–32)	21 (16–27)	**<0.001**
**ACR (mg/mmol)**	28 (6–116)	30 (6–130)	39 (8–133)	0.32
**CRP (mg/L)**	1.8 (0.9–3.9)	3.1 (1.7–4.9)	6.6 (2.5–16.7)	**<0.001**
**Serum cFLC (mg/L)**	60 (45–78)	81 (61–109)	128 (115–145)	**<0.001**
**Serum LG1M (ng/mL)**	8.5 (8.5–11.4)	21.6 (17.4–25.1)	44.5 (36.1–62.1)	**<0.001**

**Table 2:** Baseline demographic, social, and clinical characteristics of the cohort, divided into uLG1M tertiles. Data are presented as number (%) for categorical variables, while continuous variables are presented as median (IQR). DM = diabetes mellitus; COPD = chronic obstructive pulmonary disease; CVD = cerebrovascular disease; IHD = ischaemic heart disease; PVD = peripheral vascular disease; CCI = Charlson’s comorbidity index; IMD = index of multiple deprivation; BMI = body mass index; PWV = pulse wave velocity; PP = pulse pressure; CKD-EPI = Chronic Kidney Disease Epidemiology Collaboration; eGFR = estimated glomerular filtration rate; ACR = albumin creatinine ratio; CRP = C-reactive protein; cFLC = combined free light chains.

	uLG1M tertiles			
	1 [14.3–24.1]	2 [29.8–38.5]	3 [47.6–72.0]	p
n	164	163	164	
Age (years)	56 (45–68)	63 (50–76)	73 (61–81)	**<0.001**
Sex (male)	111 (67.7%)	96 (58.9%)	94 (57.3%)	0.12
Ethnicity White South Asian Black Other	109 (66.5%) 33 (20.1%) 22 (13.4%) 0 (0%)	120 (73.6%) 30 (18.4%) 11 (6.7%) 2 (1.3%)	127 (77.5%) 24 (14.6%) 10 (6.1%) 3 (1.8%)	0.50 0.48 0.05 1.00
Primary renal diagnosis Ischaemia/hypertensive nephropathy Diabetes Glomerulonephritis Polycystic kidney disease Other/uncertain	31 (21.1%) 10 (6.8%) 40 (27.2%) 11 (7.5%) 55 (37.4)	38 (25.3%) 21 (14.0%) 22 (14.7%) 15 (10.0%) 54 (36.0%)	56 (36.8%) 17 (11.2%) 21 (13.8%) 3 (2.0%) 55 (36.2%)	**0.02** 0.14 **0.02** **0.02** 0.99
Co-morbidities Malignancy DM COPD CVD IHD PVD	25 (15.2%) 34 (20.7% 14 (8.5%) 11 (6.7%) 21 (12.8%) 8 (4.9%)	26 (16.0%) 70 (43.0%) 20 (12.3%) 20 (12.3%) 39 (24.0%) 17 (10.4%)	19 (11.6%) 75 (45.7%) 25 (15.2%) 21 (12.8%) 49 (29.9%) 24 (14.6%)	0.54 **0.002** 0.21 0.17 **0.004** **0.02**
Smoking status (current smoker)	25 (15.2%)	24 (14.7)	16 (9.8%)	0.27
IMD Score	34 (16–48)	29 (18–44)	24 (17–42)	0.36
BMI (kg/m^2^)	29 (25–33)	29 (25–34)	28 (25–33)	0.48
Systolic blood pressure (mmHg)	121 (110–134)	125 (113–137)	128 (115–142)	**0.01**
Diastolic blood pressure (mmHg)	76 (69–84)	75 (68–83)	72 (65–80)	**0.002**
PWV (mmHg)	8.8 (7.7–10.3)	9.8 (8.4–11.6)	10.2 (8.7–11.7)	**<0.001**
PP (mmHg)	41 (34–53)	48 (38–64)	56 (43–69)	**<0.001**
Cystatin C (mg/L)	2.1 (1-8-2.8)	2.5 (1.8–3.0)	2.7 (2.1–3.2)	**<0.001**
CKD-EPI eGFR (mL/min/1.73m^2^)	29 (21–38)	27 (19–36)	24 (18–29)	**0.001**
ACR (mg/mmol)	17 (9–24)	28 (5–123)	22 (7–95)	0.16
CRP (mg/L)	2.6 (1.2–5.1)	2.8 (1.3–6.8)	4.0 (1.9–8.9)	**0.003**
Serum cFLC (mg/L)	69 (51–96)	76 (52–104)	86 (64–117)	**0.005**
Urine LG1M/Creatinine (ng/μmol)	19.9 (14.3–24.1)	34.8 (29.8–38.5)	56.4 (47.6–72.0)	**<0.001**

Patients in the highest sLG1M tertile were older, had a higher BMI, a higher systolic blood pressure and lower kidney function (Table 1). Patients in the highest uLG1M tertile were older, had a higher systolic blood pressure, a lower diastolic blood pressure and lower kidney function (Table 2).

Co-morbidities and their association to sLG1M and uLG1M levels are shown in Table 3. Both sLG1M and uLG1M levels were higher in patients with diabetes (p<0.001), COPD (p<0.04) and peripheral vascular disease (p<0.02). Moreover, levels of uLG1M were higher in patients with ischaemic heart disease (p<0.0001) and cerebrovascular disease (p = 0.016).

**Table 3:** Median sLG1M (ng/mL) and uLG1M in patients with different co-morbidities.

Co-morbidity	sLG1M			uLG1M		
	No	Yes	p-value	No	Yes	p-value
COPD	26.93	36.26	0.007	31.78	37.91	0.034
Ischaemic heart disease	27.80	28.92	0.483	30.51	40.12	<0.001
Cerebrovascular Disease	28.56	23.76	0.531	31.68	39.62	0.016
Peripheral Vascular Disease	27.53	32.79	0.012	37.36	50.43	0.001
Diabetes	25.28	32.88	0.001	28.88	39.83	<0.001
Malignacy	28.13	27.59	0.876	32.69	31.18	0.483

Differences in LG1M levels in patients with co-morbidities were assessed by a non parametric Mann-Whitney analysis.

**Fig 1:**
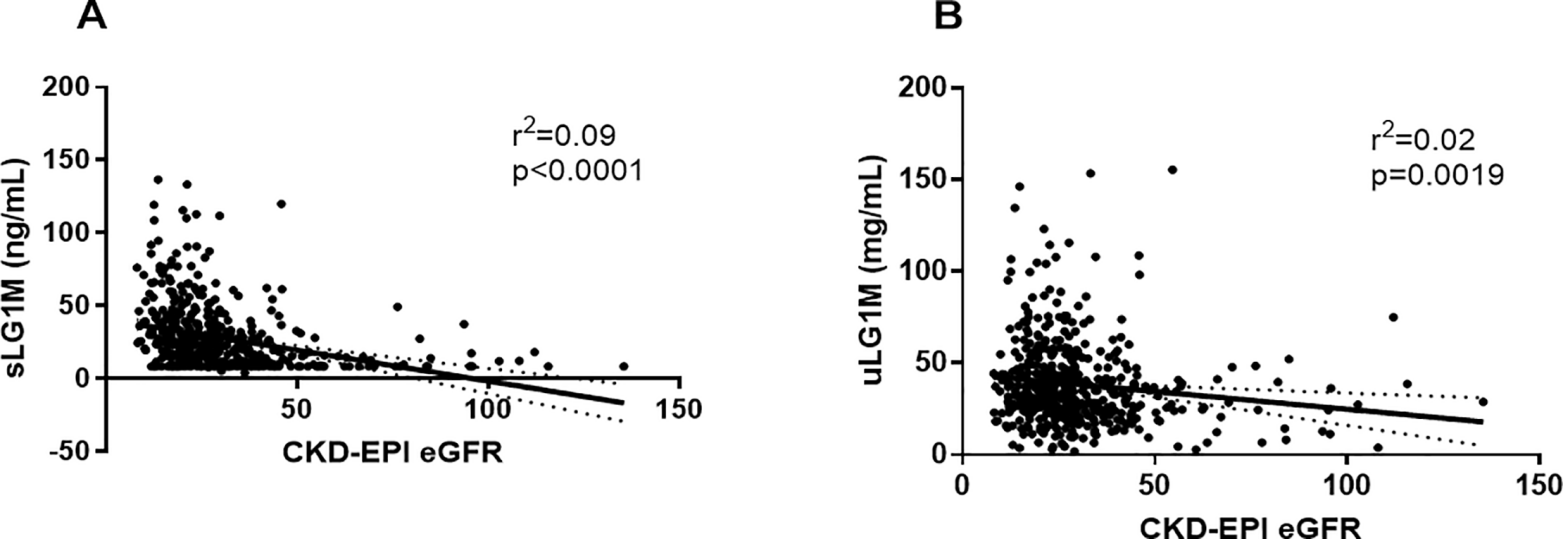
Association between renal function and sLG1M and uLG1M. Scatter plot of sLG1M (A) and uLG1M (B) by CKD-EPI eGFR. A regression line with 95% CI is shown in the graph.

A scatterplot of sLG1M and CKD-EPI eGFR showed an association of sLG1M with eGFR (r^2^ = 0.09, p<0.0001, Fig 1A) and uLG1M with eGFR (r^2^ = 0.02, p = 0.0019, Fig 1B).To assess whether the increased levels of LG1M in urine were due to proteinuria, the biomarker was correlated to ACR. There was only a very weak correlation between uLG1M and ACR (r = -0.09, p = 0.04). Serum LG1M was moderately correlated with CRP (r = 0.469, p<0.0001) and cFLC (r = 0.387, p<0.0001) while uLG1M had a very weak correlation with CRP (r = 0.160, p = 0.0006).

### One-year progression

**Fig 2:**
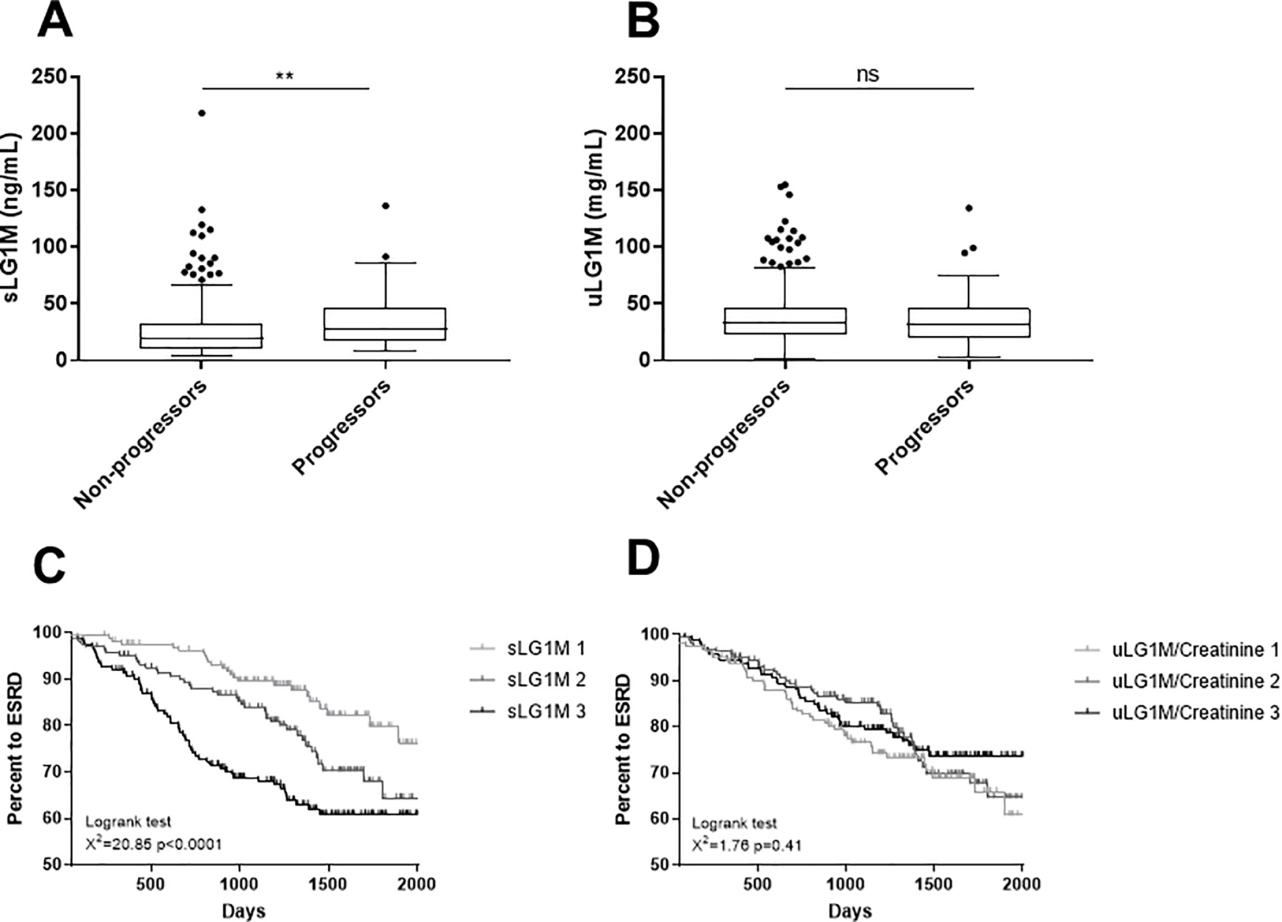
Association of sLG1M and uLG1M with 12-months disease progression, and development of ESRD. Tukey boxplots showing levels of (A) sLG1M and (B) uLG1M in patients that did not progress (non-progressors, n = 370) and progressed (progressors, n = 46) within one-year. One-year disease progression was defined as a decline in eGFR of >30% or start of RRT within one year. Using a non-parametric Mann Whitney U test we showed that sLG1M levels were significantly higher in progressors than non-progressors (A). Kaplan-Meier curves were performed for sLG1M tertiles to assess the association with development of ESRD. Patients in the highest tertile were significantly more likely to develop ESRD (p = 0.0001) (C). Kaplan-Meier curves were performed for uLG1M tertiles to assess the association with with development of ESRD. Patients in the different tertiles did not have any significant difference in risk of developing ESRD (p = 0.41) (D). Significance levels: ns = non-significant, **p<0.01.

Forty-six (11%) of 416 participants alive with one-year follow up data had progression of CKD. Baseline sLG1M was significantly elevated in progressors compared to non-progressors (p = 0.003) (Fig 2A). No significant difference was seen for uLG1M between the two groups (p = 0.56) (Fig 2B). To test the strength of this association, important confounders were tested for association to progression in a univariable cox proportional hazard regression. The variables with a univariable association with one-year progression were included in the multivariable logistic regression analysis together with sLG1M or uLG1M. The variables that were retained in the model were ACR and eGFR. Neither age, sLG1M nor uLG1M were retained in the final model and are not independently associated with one-year progression (data not shown).

### Development of end stage renal disease

One-hundred and twenty-two (24.8%) participants developed ESRD during the follow up period. Kaplan-Meier survival curves are shown for sLG1M and uLG1M (Fig 2C and 2D). While uLG1M was not prognostic for development of ESRD (HR 1.00, 95% CI 0.98–1.03, p = 0.41), patients in the highest sLG1M tertile had a 3.2 fold (HR 3.2, 95% CI 1.99–5.19) higher risk of developing ESRD compared to patients in the lowest tertile (χ^2^ = 20.85; p = 0.0001). However, after adjustment for age, ACR, and eGFR, sLG1M was no longer significant associated with the development of ESRD.

### Mortality

**Fig 3:**
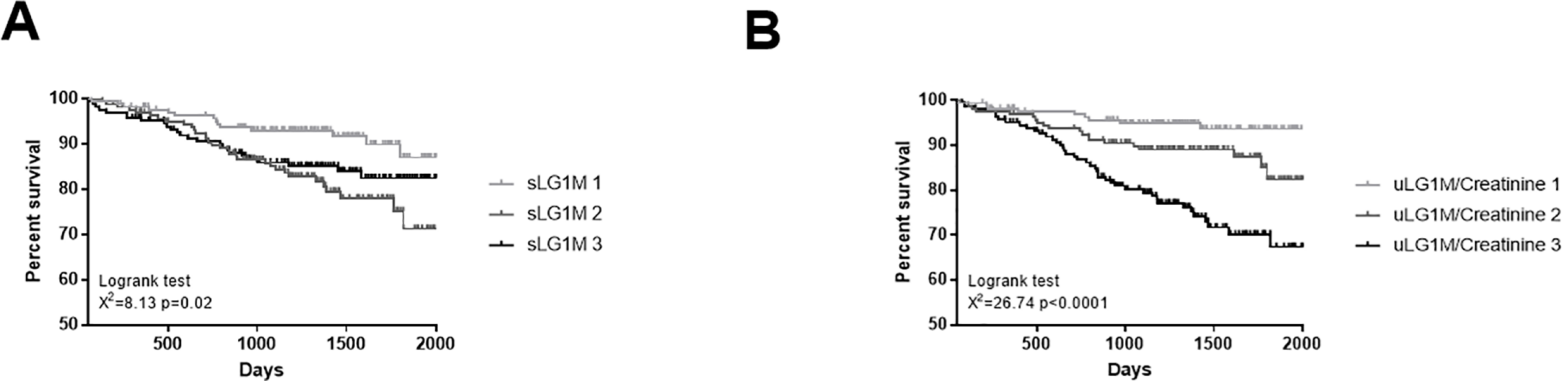
Association of sLG1M and uLG1M with mortality. **(**A) Kaplan-Meier curves were performed for sLG1M tertiles to assess the association with mortality. Patients in the highest tertile were significantly more likely to die (p = 0.02). (B) Kaplan-Meier curves were performed for uLG1M/Creatinine tertiles to assess the association with mortality. Patients in the highest tertile were significantly more likely to die (p<0.0001).

During the follow up period 71 (14.5%) participants died. Patients in the highest sLG1M tertile had a 1.8-fold increased risk of death compared to those in the lowest sLG1M tertile (HR 1.8, 95% CI 1.1–3.2, p = 0.02) and patients in the highest uLG1M tertile had a 5-fold increased risk of death compared to those in the lowest uLG1M tertile (HR 5.0, 95% CI 2.8–8.9, p<0.0001). The number and percentage of patients in each sLG1M tertiles who died were 14 (8.5%, T1), 32 (19.6%, T2) and 25 (15.3%, T3) (χ^2^ = 8.1, p = 0.02), while the number and percentage of patients in each uLG1M tertiles who died were 9 (5.5%, T1), 20 (12.2%, T2) and 42 (25.8%, T3) (χ^2^ = 26.74, p<0.0001). Kaplan-Meier survival curves for sLG1M and uLG1M are shown in Fig 3A and 3B.

In multivariable analyses, uLG1M was retained in the final model (HR 1.01, 95% CI 1.00–1.02, p = 0.002) along with age and IHD, indicating that uLG1M is independently associated with mortality (Table 4). sLG1M was not retained in the final model.

**Table 4:** Multivariable Cox regression model for mortality. Data are presented as hazard ratio (95% CI). eGFR = estimated glomerular filtration rate; ACR = albumin creatinine ratio, DM = diabetes mellitus, IHD = ischaemic heart disease, PVD = perivascular disease, CRP = c-reactive protein, uLG1M = urinary LG1M/Creatinine.

Variable	Hazard Ratio (95% CI)	p
**Age**	1.07 [1.04–1.09]	**<0.0001**
**CKD-EPI eGFR^a^**	1.21 [0.82–1.79]	0.327
**ACR^a^**	1.00 [1.00–1.01]	0.377
**DM**	0.92 [0.54–1.57]	0.750
**IHD**	1.86 [1.09–3.17]	**0.023**
**PVD**	0.65 [0.30–1.39]	0.264
**CRP**	1.00 [0.99–1.02]	0.796
**uLG1M^a^**	1.01 [1.01–1.02]	**0.002**

^a^; Per unit.

## Discussion

In the present study, we have developed and evaluated a novel immunoassay targeting LG1M, a neo-epitope fragment of LAMC1, as a potential prognostic marker of progression and mortality in high-risk CKD patients. This marker can be measured in both serum and urine, yielding different results in the two matrices.

Utilizing the assay in a prospective cohort of patients with CKD at high risk of progression we found that; 1) both sLG1M and uLG1M increased with CKD severity, 2) sLG1M was elevated in patients that progressed over one year of follow-up, but not as an independent risk factor, and 3) uLG1M was independently associated with mortality in high-risk CKD patients.

Laminin is one of the main constituents of the basement membrane. Laminin-11, containing LAMC1, contributes to the network required to maintain the integrity of the glomerular basement membrane[21,22]. LAMC1 staining intensity in the glomerular basement membrane was shown to be increased in immunohistochemical analysis of kidney specimens from patients with CKD[21].

The developed LG1M assay detects a degradation fragment of LAMC1 which is released by MMP-9, an MMP known to be highly upregulated in fibrosis[23]. Therefore, this assay can be used to non-invasively describe the dynamic process of basement membrane remodeling occurring during fibrosis.

The developed assay showed high sensitivity towards the target and analytical robustness. The assay satisfied the acceptance requirements in all technical aspects, showing a good linearity and reproducibility.

The cohort analyzed in this study consisted of high-risk CKD patients. All patients had moderate to severe impairment of kidney function and it is known that kidney fibrosis is present in these stages of CKD[24–26]. High levels of LG1M in serum and urine were both related to CKD severity and to disease progression and mortality in this study. Since LG1M is generated upon basement membrane degradation, this finding may suggest that LG1M reflects the remodeling of the glomerular and tubular basement membrane that characterize glomerulosclerosis and tubulointerstitial fibrosis and drive disease progression[27].

The γ1 chain of laminin is expressed in the majority of the laminin isoforms, and it is therefore ubiquitously present in basement membranes throughout the body. Since we saw that levels of LG1M in urine were associated with ischaemic heart disease, cerebrovascular disease, and peripheral vascular disease, we cannot exclude that there was a contribution from the vascular basement membrane to the pool of LG1M fragments in urine.

We have observed that levels of LG1M in serum were related to renal-associated outcomes. Whilst not retained in the final model for prediction of progression to ESRD, levels of sLG1M were more elevated in patients that progressed over one year of follow-up, and patients in the highest tertile of sLG1M were three times more likely to progress to ESRD than the patients in the lowest tertile. Moreover, we observed an association of sLG1M with CRP and sFLC, which have both been shown to be associated with adverse outcomes in CKD[28–32]. Therefore, sLG1M could be a surrogate biomarker for systemic factors that contribute to progression of kidney disease.

Conversely, levels of LG1M in urine were related to all-cause mortality, and the association of the marker with mortality was strongly maintained even after adjustments for important confounders for mortality, including eGFR and albuminuria. This may indicate that changes in basement membrane remodeling contribute to pathological processes that lead to mortality in CKD patients.

The strength of this study is the use of a large prospective cohort of patients with high-risk CKD to validate the association of the novel biomarker LG1M in both serum and urine with relevant clinical end-points. The limitations of this study include the use of a single-centre cohort; the lack of a mechanistic explanation for the association between LG1M and the clinical outcomes; and the fact that LAMC1 is present in several organs throughout the body, and therefore cannot be qualified as kidney-specific. Moreover, due to the fact that kidney biopsies were not collected from these patients, we were unable to do a direct comparison of levels of LG1M in urine and serum with the extent of renal fibrosis. Further studies on CKD populations that underwent renal biopsy are required to establish a direct link between this marker of laminin remodeling and burden and progression of renal fibrosis.

In conclusion, this study shows that LG1M, a marker of basement membrane remodeling, is elevated in serum of one-year CKD progressors and it is a risk factor for development of ESRD when measured in serum, and is independently associated with mortality when measured in urine of high-risk CKD patients.

## Supporting information

S1 Fig Sequence alignment between human, mouse and rat laminin-gamma-1 chain. The antibody recognizes the residues from 1232 to 1241, which are 100% homologous between human, mouse and rat.
(TIF)

S2 Fig Assay specificity. Reactivity to the standard peptide (LNRKYEQAKN), the elongated peptide (LNRKYEQAKNI) and a nonsense peptide (GGPGFGPGVV) was tested for the assay LG1M. The peptide concentrations were started at 500 ng/mL, and diluted as a 2-fold dilution. The background signal from the system was tested using a nonsense coating peptide (Biotin-LNRKYEQAKN). The data are presented as percentage (%) of background absorbance, which is the absorbance of the assay buffer, as a function of peptide concentration.
(TIF)

S3 Fig LG1M ELISA runs showing typical standard curves and native reactivity against A) human urine, B) human serum, C) mouse serum, rat serum, D) mouse urine and rat urine. The standard peptide was 2-fold diluted starting from 500 ng/mL. The samples were run from undiluted and up to 8-fold dilution as indicated. The data are presented as percentage (%) of background absorbance, which is the absorbance of the assay buffer, as a function of peptide concentration.
(TIF)

S1 Table Percentage dilution recovery for the serum and urinary LG1M assay using human serum (HS), human urine (HU), rat serum (RS), rat urine (RU), mouse serum (MS) and mouse urine (MU). (DOCX)

S2 Table Technical validation of the LG1M assay. (DOCX)

S1 File (XLSX)

S1 Text (DOCX)
