# Supplementary figures and images for: A novel biomarker of laminin turnover is associated with disease progression and mortality in chronic kidney disease
Source: PLoS One. 2018 Oct 1;13(10):e0204239. doi: 10.1371/journal.pone.0204239 (PMC6166934; doi:10.1371/journal.pone.0204239)

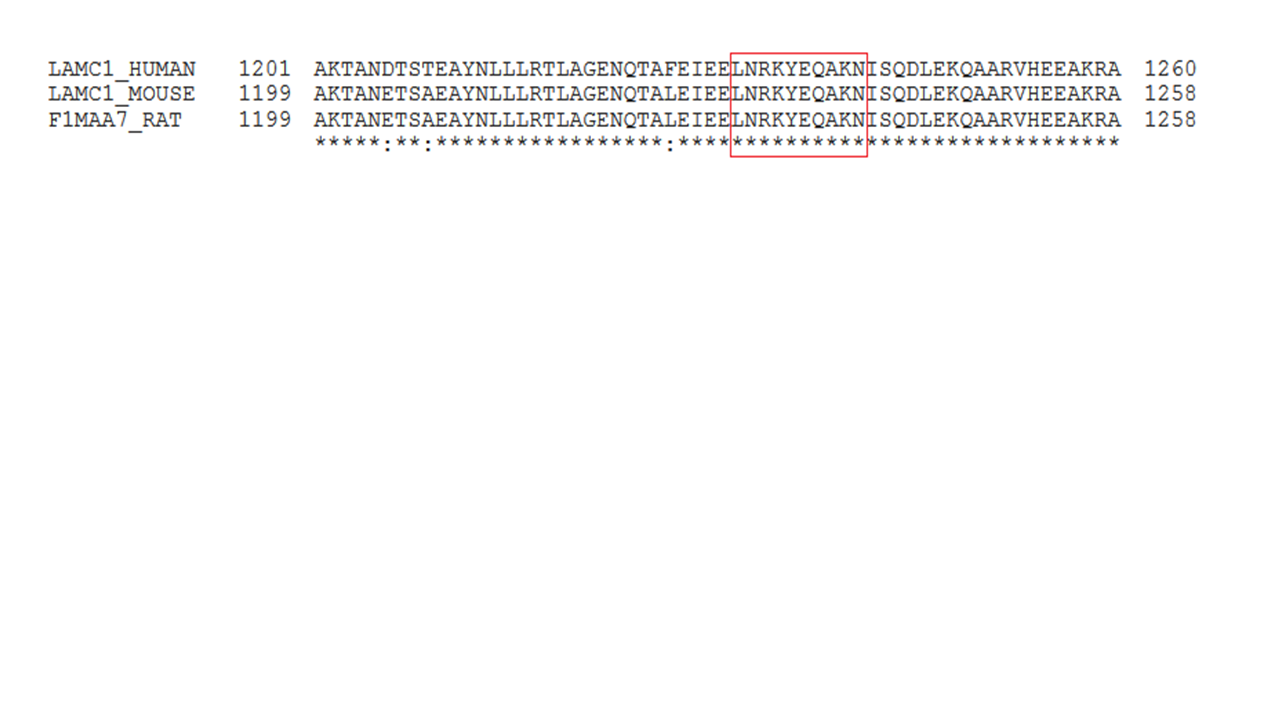

Supplement: S1 Fig — The antibody recognizes the residues from 1232 to 1241, which are 100% homologous between human, mouse and rat. (TIF) [file pone.0204239.s001.tif]

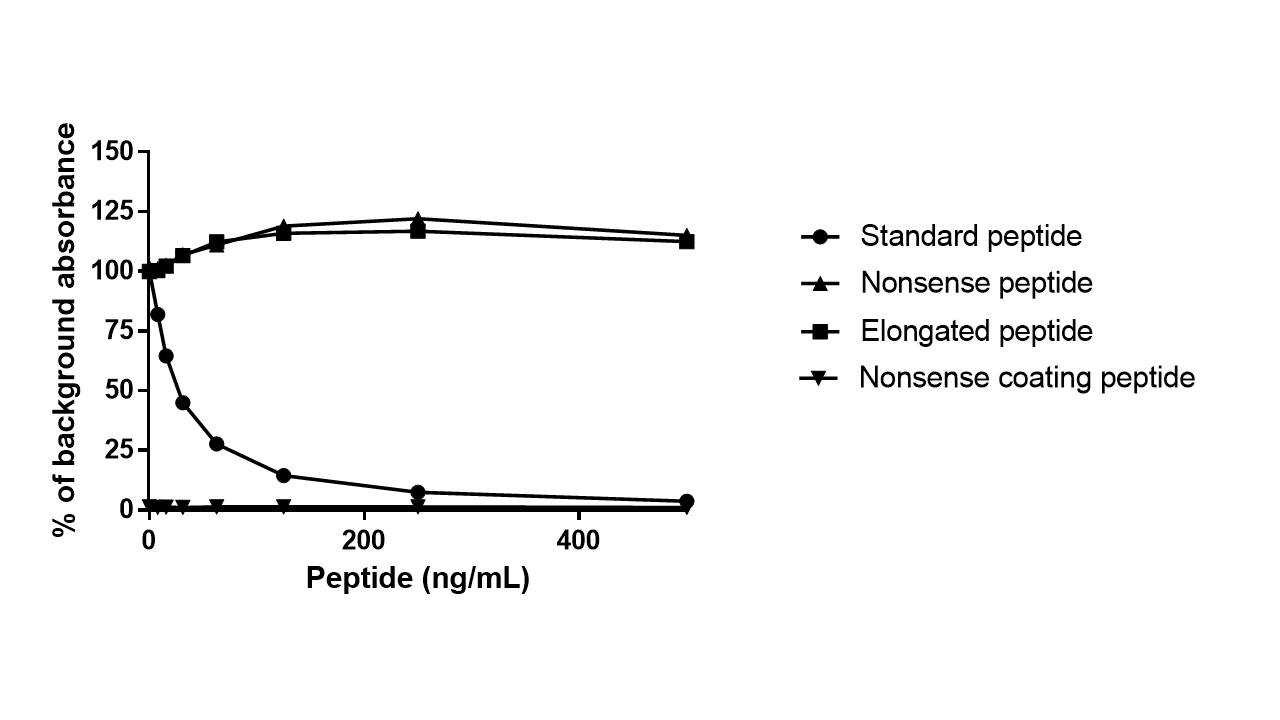

Supplement: S2 Fig — Reactivity to the standard peptide (LNRKYEQAKN), the elongated peptide (LNRKYEQAKNI) and a nonsense peptide (GGPGFGPGVV) was tested for the assay LG1M. The peptide concentrations were started at 500 ng/mL, and diluted as a 2-fold dilution. The background signal from the system was tested using a nonsense coating peptide (Biotin-LNRKYEQAKN). The data are presented as percentage (%) of background absorbance, which is the absorbance of the assay buffer, as a function of peptide concentration. (TIF) [file pone.0204239.s002.tif]

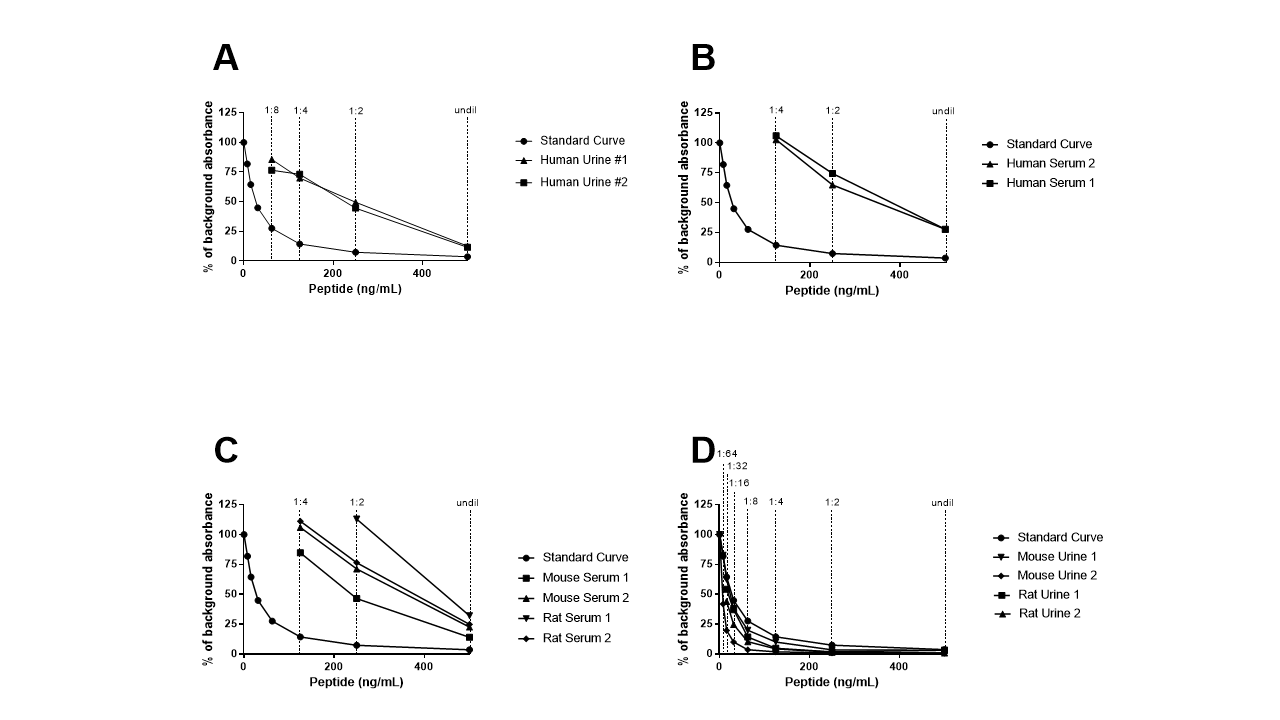

Supplement: S3 Fig — LG1M ELISA runs showing typical standard curves and native reactivity against A) human urine, B) human serum, C) mouse serum, rat serum, D) mouse urine and rat urine. The standard peptide was 2-fold diluted starting from 500 ng/mL. The samples were run from undiluted and up to 8-fold dilution as indicated. The data are presented as percentage (%) of background absorbance, which is the absorbance of the assay buffer, as a function of peptide concentration. (TIF) [file pone.0204239.s003.tif]
