# Supplementary material for: A novel biomarker of laminin turnover is associated with disease progression and mortality in chronic kidney disease
Source: PLoS One. 2018 Oct 1;13(10):e0204239. doi: 10.1371/journal.pone.0204239 (PMC6166934; doi:10.1371/journal.pone.0204239)
Supplement: S1 Table — (DOCX) [file pone.0204239.s004.docx]

**S1 Table.** Percentage dilution recovery for the serum and urinary LG1M assay using human serum (HS), human urine (HU), rat serum (RS), rat urine (RU), mouse serum (MS) and mouse urine (MU).

| LG1M (ng/mL) | HS (n=4) | HU (n=4) | RS (n=2) | RU (n=2) | MS (n=2) | MU (n=2) |
| --- | --- | --- | --- | --- | --- | --- |
| Undiluted | 113% | 90% | 75% | 73% | 83% | 88% |
| Dilution 1:2 | 87% | 100% | 80% | 94% | 88% | 83% |
| Dilution 1:4 | - | 107% | - | - | - | 92% |
| Mean | 100% | 99% | 78% | 86% | 85% | 88% |
