# Supplementary material for: A novel biomarker of laminin turnover is associated with disease progression and mortality in chronic kidney disease
Source: PLoS One. 2018 Oct 1;13(10):e0204239. doi: 10.1371/journal.pone.0204239 (PMC6166934; doi:10.1371/journal.pone.0204239)
Supplement: S2 Table — (DOCX) [file pone.0204239.s005.docx]

**S2 Table. Technical validation of the LG1M assay.**

| Technical validation test | LG1M |
| --- | --- |
| Detection range | 8.5-279.0 ng/mL |
| Intra-assay variation | 8.8 % |
| Inter-assay variation | 14.9 % |
| Dilution recovery in human serum^1^ | 100% (87-113%) |
| Dilution recovery in rat serum^1^ | 78% (75-80%) |
| Dilution recovery in mouse serum^1^ | 85% (83-88%) |
| Dilution recovery in human urine^1^ | 99% (90-107%) |
| Dilution recovery in rat urine^1^ | 86% (73-94%) |
| Dilution recovery in mouse urine^1^ | 88% (83-92%) |
| Analyte stability 24h, 4^0^C/20^0^C | 118% (115-120%) |
| Hemoglobin interference, low/high | 102% / 99% |
| Lipemia interference, low/high | 125% / 92 % |
| Biotin interference, low/high | 72% / 61% |
| Salt interference, pH 6.0/pH 7.0/pH 8.0^2^ | 97 % |
| Spiking recovery (peptide in serum) | 50% |
| Spiking recovery (peptide in urine) | 87.7% |

^1^Percentages are reported as mean with range shown in brackets, ^2^Average salt interference
