# Supplementary material for: A novel biomarker of laminin turnover is associated with disease progression and mortality in chronic kidney disease
Source: PLoS One. 2018 Oct 1;13(10):e0204239. doi: 10.1371/journal.pone.0204239 (PMC6166934; doi:10.1371/journal.pone.0204239)
Supplement: S1 Text — (DOCX) [file pone.0204239.s007.docx]

**Supplementary material**

**Supplementary materials and methods**

**Monoclonal antibody**

The amino acid sequence 1232’-LNRKYEQAKN-’1241 for the laminin gamma-1 chain is 100% homologous between human, mouse and rat (S1 Fig). This fragment was identified as a novel cleavage fragment of MMP-9 in the C-terminus by nano LC-MS-MS as previously described^1^. Generation of monoclonal antibodies was performed as previously described^2^. Briefly, production was initiated by subcutaneous immunization of 4-5 week old Balb/C mice with 200 μl emulsified antigen and 50 μg immunogenic peptide (ie, KLH-CGG-LNRKYEQAKN) using Freund’s incomplete adjuvant. The immunizations were repeated every second week until stable serum titer levels were reached. The mouse with the highest serum titer, was then rested for a month and then boosted intravenously with 50 μg immunogenic peptide in 100 μl 0.9% NaCl solution three days before isolation of the spleen. The spleen cells were fused with SP2/0 myeloma cells to produce a hybridoma as described by Gefter and co-workers [17] and cloned in culture dishes using the semi-solid medium method. The clones were plated into 96-well microtiter plates for further growth employing the limited dilution method to secure monoclonal growth. The supernatants were screened for reactivity against standard peptide and native material in an indirect ELISA using streptavidin-coated plates. Biotin-LNRKYEQAKN was used as screening peptide, while the standard peptide LNRKYEQAKN was used as a calibrator to test for further specificity of clones. The mice were housed under standardized conditions (20–23 °C, 30–60% relative humidity, and a 12-h/12-h light/dark cycle) and were caged five mice per cage in cage type type lllH (1291H, 425x266x185mm, floor area 800cm2) from Scanbur DK. They had access to food and water ad libitum, and the general conditions were monitored daily by trained animal technicians. To minimize the stress, the mice were only handle when necessary during dosing and when changing the cages. The mice also had access to materials for making nest and lay calmly by having access to Inviro-DRI (31008), mouse house/mouse igloo (Scanbur), Tapvai 2HV (37311), Aspe wooden stick S (30968) and NESTLETS (31007) from Brogaarden DK. When the analgesics is giving, it was only by trained Animal technicians.  Mice was sacrificed by cervical dislocation. The study was approved by the Danish Animal Experimentation council (J. Nr.: 2013-15-2934-00956) and conducted according to national legislation.

**Clone characterization**

Native reactivity and peptide affinity for the standard peptide were assessed using human serum and human urine purchased from a commercial supplier (Valley Biomedical, VA 22602, USA). Rat and mouse urine and serum was obtained from 3 months old healthy Sprague-Dawley rats and Balb/c mice, housed at Nordic Bioscience. They were sacrificed by cervical dislocation. The study was approved by the Danish Animal Experimentation council (J. Nr.: 2016-15-0201-00933) and conducted according to national legislation. Antibody specificity was tested in a preliminary assay using deselection and elongated peptides (i.e., standard peptide with ten amino acid substitutions and standard peptide with one additional amino acid at the cleavage site (LNRKYEQAKNI, respectively). The isotype of the monoclonal antibody was determined using the Clonotyping System-HRP kit, cat. 5300-05 (Southern Biotech, Birmingham, AL, USA).

**LG1M ELISA**

Supernatant from antibody-producing hybridoma was collected and the monoclonal antibody was purified using HiTrap affinity columns (GE Healthcare Life Science, Little Chalfront, Buckinghamshire, UK) and labeled with horseradish peroxidase (HRP) using Lightning-Link^TM^ HRP Conjugation Kit (Innova Biosciences, Babraham, Cambridge, UK), according to the manufacturer’s instructions.

The final LG1M competitive ELISA procedure was as follows; A 96-well streptavidin-coated plates were incubated with 5 ng/mL biotinylated-peptide for 30 min at 20 °C with shaking at 300 rpm. Plates were washed five times in washing buffer (20 mM TRIS, 50 mM NaCl, pH 7.2). Sample/standard/control (20 µl) was added and followed immediately by addition of 220 ng/mL HRP labeled peptide-specific monoclonal antibody and incubated for 3h at 4 °C with shaking at 300 rpm. After incubation, plates were washed five times in washing buffer. Finally, 100 µl 3,3’,5,5’-Tetramethylbenzidine (TMB, Kem-En-Tec Diagnostics) was added and incubated for 15 min at 20 °C in the dark with shaking at 300 rpm. To stop the enzyme reaction of TMB, 100 µl 0.1% sulphuric acid was added and the plate was analyzed in the ELISA reader at 450 nm with 650 nm as the reference (Molecular Devices, SpectraMax M, CA, USA). A standard curve was plotted using a 4-parametric mathematical fit model. Each ELISA plate included kit controls to monitor inter-assay variation. All samples were measured within the measurement ranges of the assay.

**Technical evaluation**

A 2-fold dilution of human serum and human urine was used to determine linearity, calculated as percentage of recovery of the undiluted sample. Antibody specificity was calculated as percentage of signal inhibition by 2-fold diluted standard peptide (LNRKYEQAKN), elongated peptide (LNRKYEQAKNI), and non-sense peptide (GGPGFGPGVV). Lower limit of detection (LLOD) was calculated as the mean + 3*Standard Deviation (SD) of the blank from 21 determinations of assay buffer. Upper limit of detection (ULOD) was determined as the mean – 3*SD of 10 measurements of the highest point on the standard curve (standard A). The intra- and inter-assay variation was determined by 10 independent measurements of seven quality control samples in double determination. Accuracy of the assay was measured in healthy human serum/urine samples spiked with standard peptide and a serum/urine sample with a known high LG1M concentration, and calculated as the percentage recovery of serum/urine in buffer. Interference was measured in healthy human serum spiked with hemoglobin (low=0.155 mM, high=0.310 mM), lipids (low=4.83 mM, high=10.98 mM), and biotin (low=30 ng/ml, high=90 ng/ml) and calculated as the percentage recovery of analyte in non-spiked serum. Interference with salt samples (8.14 g/L) was tested at pH 6.0, pH 7.0 and pH 8.0 to ensure no reactivity towards high salt content in urine, and changes in pH values of urine. The analyte stability was determined for two human sera and one human urine in four freeze-thaw cycles. Recovery was calculated with the first cycle as reference value.

**Supplementary results**

**Clone selection and characterization**

The monoclonal antibody with the best native reactivity towards the standard peptide and native material was chosen from the antibody-producing clones generated after fusion between the mouse spleen cells and myeloma cells. Based on reactivity, we selected the antibody clone NB310-2, which was determined to be an IgG1 subtype. No inhibition was observed using the elongated peptide and non-sense peptide, indicating specificity of the antibody for the neo-epitope sequence (S2 Fig).

**Technical evaluation of LG1M**

The measurement range of the LG1M ELISA was determined by calculating the lower limit of detection (LLOD) and upper limit of detection (ULOD), which provided a measurement range of 8.5 to 279.0 ng/mL.
The mean dilution recovery for human serum and human urine was close to 100% for undiluted to 1:4, while mouse and rat serum could only be diluted to 1:2, since further dilutions caused the signal to fall below LLOD. Mouse urine needed to be diluted between 1:16 and 1:32, while rat urine needed to be diluted from 1:8 to 1:32 in order to be measurable within the measurement range of the assay (See S3 Fig). The dilutions for the individual species can be found in S1 Table. At the stated dilutions, all recoveries were within the acceptable range of 100±20%. The analyte stability was acceptable for four freeze/thaw cycles of human serum and human urine (S2 Table). Spiking recovery for standard peptide in urine was acceptable (S2 Table). Interference tests using hemoglobin, lipids, and salt showed that these substances did not generate interference when added to the assays at high concentrations; however, high levels of biotin interfered with the assay (S2 Table). These levels are though sensibly exceeding the concentrations normally present in human serum. Intra- and inter-assay variation showed acceptable values of 8.8% and 14.88%, respectively (S2 Table).

Native reactivity was observed in human serum, human urine, rat serum, rat urine, mouse serum and mouse urine (See S3 Fig).

**Supplementary Figures**

**S1 Fig.** Sequence alignment between human, mouse and rat laminin-gamma-1 chain. The antibody recognizes the residues from 1232 to 1241, which are 100 % homologous between human, mouse and rat.

**S2 Fig. Assay specificity.** Reactivity to the standard peptide (LNRKYEQAKN), the elongated peptide (LNRKYEQAKNI) and a nonsense peptide (GGPGFGPGVV) was tested for the assay LG1M. The peptide concentrations were started at 500 ng/mL, and diluted as a 2-fold dilution. The background signal from the system was tested using a nonsense coating peptide (Biotin-LNRKYEQAKN). The data are presented as percentage (%) of background absorbance, which is the absorbance of the assay buffer, as a function of peptide concentration.

**S3 Fig.** LG1M ELISA runs showing typical standard curves and native reactivity against A) human urine, B) human serum, C) mouse serum, rat serum, D) mouse urine and rat urine. The standard peptide was 2-fold diluted starting from 500 ng/mL. The samples were run from undiluted and up to 8-fold dilution as indicated. The data are presented as percentage (%) of background absorbance, which is the absorbance of the assay buffer, as a function of peptide concentration.

**Supplementary References**

1. Thingholm TE, Larsen MR. The Use of Titanium Dioxide Micro-Columns to Selectively Isolate Phosphopeptides from Proteolytic Digests. *Methods in molecular biology (Clifton, N.J.)* 2009. p. 57–66.

2. Leeming DJ, Nielsen MJ, Dai Y, Veidal SS, Vassiliadis E, Zhang C, He Y, Vainer B, Zheng Q, Karsdal M a. Enzyme-linked immunosorbent serum assay specific for the 7S domain of Collagen Type IV (P4NP 7S): A marker related to the extracellular matrix remodeling during liver fibrogenesis. *Hepatol Res* 2012;**42**:482–493.
